# High-Value Utilization of Silicon Cutting Waste and Excrementum Bombycis to Synthesize Silicon–Carbon Composites as Anode Materials for Li-Ion Batteries

**DOI:** 10.3390/nano12162875

**Published:** 2022-08-21

**Authors:** Hengsong Ji, Jun Li, Sheng Li, Yingxue Cui, Zhijin Liu, Minggang Huang, Chun Xu, Guochun Li, Yan Zhao, Huaming Li

**Affiliations:** 1 Institute for Energy Research, Jiangsu University, Zhenjiang 212013, China; 2 Key Laboratory of Fine Chemical Application Technology of Luzhou, Luzhou 646099, China; 3 College of Chemistry and Chemical Engineering, Inner Mongolia University, Hohhot 010021, China

**Keywords:** silicon–carbon composites, silicon cutting waste, excrementum bombycis, biomass, anode materials, lithium-ion batteries

## Abstract

Silicon-based photovoltaic technology is helpful in reducing the cost of power generation; however, it suffers from economic losses and environmental pollution caused by silicon cutting waste. Herein, a hydrothermal method accompanied by heat treatment is proposed to take full advantage of the photovoltaic silicon cutting waste and biomass excrementum bombycis to fabricate flake-like porous Si@C (FP-Si@C) composite anodes for lithium-ion batteries (LIBs). The resulting FP-Si@C composite with a meso-macroporous structure can buffer the severe volume changes and facilitate electrolyte penetration. Meanwhile, the slightly graphitic carbon with high electrical conductivity and mechanical strength tightly surrounds the Si nanoflakes, which not only contributes to the ion/electron transport but also maintains the electrode structural integrity during the repeated lithiation/delithiation process. Accordingly, the synergistic effect of the unique structure of FP-Si@C composite contributes to a high discharge specific capacity of 1322 mAh g^−1^ at 0.1 A g^−1^, superior cycle stability with a capacity retention of 70.8% after 100 cycles, and excellent rate performance with a reversible capacity of 406 mAh g^−1^ at 1.0 A g^−1^. This work provides an easy and cost-effective approach to achieving the high-value application of photovoltaic silicon cutting waste, as well as obtaining high-performance Si-based anodes for LIBs.

## 1. Introduction

Energy shortfall and environmental issues have been commonly considered as major challenges to humanity, leading to the flourishing growth of renewable energy sources [1]. Currently, the development of silicon-based photovoltaic technology is conducive to keeping the power generation cost down [2]. However, in the current photovoltaic industry, ca. 35% of solar-grade silicon ingots have to be cut into silicon cutting waste, resulting in more than 2.8 billion USD in losses annually in China [3,4]. Besides these, flake-like silicon particles of ca. 1 µm size easily cause severe water and soil contamination [5]. Therefore, the effective recovery and reuse of tons of silicon cutting waste are becoming key challenges. So far, various strategies for recycling silicon cutting waste have sprung up, such as the preparation of silicon nitride [6], Al–Si alloys [5], and surface-modified adsorbents for heavy metal ion removal from water [7]. In addition, silicon-based materials as anodes for lithium-ion batteries (LIBs) are also a valuable pathway for silicon cutting waste recovery due to their high theoretical specific capacity [8]. However, the major shortcomings of silicon-based anodes are their low electrical conductivity and huge volume changes during the lithiation/delithiation process, leading to electrode cracking and the loss of electrical contact between current collectors and active materials [9,10]. Meanwhile, the instability of solid electrolyte interphase (SEI) layers induced by the pulverization of silicon-based electrodes can cause rapid electrolyte depletion [11,12]. As a result, all of the above problems would give rise to unsatisfactory cycle and rate performance, which have restricted the development and practical application of silicon-based anodes.

In the past decades, numerous strategies have been adopted to improve the electrochemical performance of silicon-based anodes [13,14,15,16,17,18,19,20,21,22,23,24]. Typically, the approach has been the utilization of nanoscale silicon particles, which can strengthen the endurance to large volume changes, reduce the ion transport distance, and increase the contact area between silicon-based anodes and electrolyte [13]. However, the high surface energy, unsatisfactory electroconductivity, and high surface area of silicon nanoparticles can cause severe agglomeration, low tap density, and repeated formation of SEI films, which deteriorates their ion/electron transport kinetics, volumetric energy density, Coulombic efficiency (CE), and cycling performance [14]. In this regard, the addition of carbonaceous matrixes such as amorphous carbon [15,16], graphitic carbon [17,18], carbon nanotubes [19,20,21], carbon nanofibers [22,23], carbon cloth [24], graphene [25,26], and graphene oxide [27,28] is considered to be an effective method to improve electrical conductivity, enhance mechanical flexibility, and stabilize SEI formation. Compared to amorphous carbon, graphitic carbon can not only deliver higher electroconductibility but also facilitate the transport of Li^+^ into silicon nanoparticles. Unfortunately, the synthesis of these silicon–graphitic carbon composites always encounters some difficulties, including harsh reaction conditions, complex synthesis procedures, and expensive raw materials [17].

Herein, we develop a facile and low-cost method for the preparation of flake-like porous Si@C (FP-Si@C) composites for high-performance anodes in LIBs using the silicon cutting waste from the photovoltaic industry and the biomass excrementum bombycis. The as-obtained FP-Si@C composite presents a porous structure, which can accommodate the volume changes and facilitate electrolyte penetration. Meanwhile, a highly conductive layer can be constructed by wrapping slightly graphitic carbon on the surface of silicon nanoflakes, which not only contributes to the ion/electron transport but also maintains the electrode structural integrity during the repeated lithiation/delithiation process. Benefiting from the unique structure, the FP-Si@C composite exhibits excellent rate capacity, long cycling life, and outstanding CE. Remarkably, this work not only provides an easy and cost-effective approach to achieving the high-value application of photovoltaic silicon cutting waste but also can be extended to advance other related element materials for energy conversion and storage.

## 2. Materials and Methods

### 2.1. Material Synthesis

Pretreatment of the silicon cutting waste (P-SiCW): The raw silicon cutting waste agglomerations (1–100 µm, Jinko Photovoltaic Power Co., Ltd., Shanghai, China) were first put into a ball mill tank containing anhydrous ethanol and milled at 600 rpm for 4 h. After that, the nano-silicon was washed with hydrochloric acid (0.1 M), deionized water, and ethanol, respectively, to remove the organic residuals, then dried at 60 °C overnight. Finally, the flake-like nano-silicon was obtained by heating at 600 °C for 1 h under nitrogen flow.

Pretreatment of the excrementum bombycis (PEB): The pretreatment of the excrementum bombycis was similar to that of cutting waste silicon except for the calcination step.

Synthesis of flake-like porous Si@C composites (FP-Si@C): P-SiCW (133.3 mg) and PEB (266.7 mg) were dispersed into deionized water (30 mL). After stirring for 1 h, the mixture was transferred into a 50 mL Teflon container and maintained at 180 °C for 6 h. Afterward, the composites were dried at 60 °C overnight, then transferred to a tube furnace under a nitrogen atmosphere and heated at 700 °C for 1 h. The resulting flake-like porous Si@C composite was called FP-Si@C-2. FP-Si@C-1 and FP-Si@C-3 were prepared by changing the mass ratio of the P-SiCW and PEB to 1:1 and 1:3, respectively.

### 2.2. Characterization

The morphologies of the samples were observed by scanning electron microscopy (SEM, JEOL JSM-7800, Tokyo, Japan) and transmission electron microscopy (TEM, JEOL JEM-2100, Tokyo, Japan). The crystalline structures of the products were examined using X-ray diffraction (XRD, Bruker D8 ADVANCE, Berlin, German) with Cu K*α* radiation. Raman spectra were characterized on a Raman system (Jobin Yvon HR800, Paris, France) with a 532 nm laser. The carbon content in the composites was determined by employing thermogravimetric analysis (TGA, Netzsch STA 449 F3, Jupiter, Selb, Germany). The amount of silicon in the P-SiCW was measured using an X-ray fluorescence spectrometer (XRF, THERMO FISHER SCIENTIFIC ARL ADVANT’X IntelliPowerTM-4200, Waltham, MA, USA). The surface composition of the sample was investigated using X-ray photoelectron spectroscopy (XPS, Thermo FISHER SCIENTIFIC K-Alpha Nexsa, Waltham, MA, USA). The N_2_ adsorption–desorption isotherms were tested by a gas adsorption analyzer (Micrometric TriStar II 3020, Cumming, GA, USA). The specific surface area and pore size distribution was calculated by the Brunauer–Emmett–Teller (BET) method and Barrett–Joyner–Halenda (BJH) model, respectively.

### 2.3. Electrochemical Measurements

The working electrodes were prepared by mixing the active materials, Ketjen black (KB), and polyvinylidene fluoride (PVDF) binder at a weight ratio of 8:1:1 in N-methylpyrrolidone (NMP). After stirring for 1 h, the homogeneous slurries were obtained and pasted onto the copper foil current collectors and then dried at 60 °C under vacuum for 12 h. After that, the above copper foils were roll-pressed at a pressure of 10 MPa and punched into disks with a diameter of 12 mm. The mass loading of the active materials was about 1.0 mg cm^−2^. The CR2032 coin-type cells were assembled in an argon-filled glovebox using Li foils, polypropylene membranes (Celgard 2400, Charlotte, NC, USA), and 1.0 M LiPF_6_ in ethylene carbonate (EC)/dimethyl carbonate (DMC) (1:1 in volume ratio) as the counter electrodes, separators, and electrolyte, respectively. The galvanostatic charging/discharging (GCD) and cyclic voltammetry (CV) tests were conducted on the Neware battery testing system (CT-4008, Shenzhen, China) and Gamry electrochemical workstation (INTERFACE1000E, Warminster, PA, USA) in a voltage window of 0.01–1.50 V (vs. Li/Li^+^), respectively. The electrochemical impedance spectroscopy (EIS) was performed using a Gamry electrochemical workstation (INTERFACE1000E, Warminster, PA, USA) with a frequency range from 100 kHz to 10 mHz at an open circuit potential with an AC perturbation of 10 mV. The galvanostatic intermittent titration technique (GITT) measurements were tested using a Neware battery testing system (CT-4008, Shenzhen, China) at a current density of 0.1 A g^−1^ and a pulse time of 10 min between 1 h rest intervals.

## 3. Results and Discussion

As illustrated in Figure S1, the FP-Si@C composites were generated from the silicon cutting waste and excrementum bombycis biowaste. Firstly, the microsized raw silicon cutting waste agglomerations (Figure S2a) were ball-milled, washed with hydrochloric acid, and heat-treated under nitrogen flow to obtain the nanosized flake-like P-SiCW (Figure S2b). The X-ray fluorescence spectrometry (XRF) results (Table S1) reveal that Si is the main component in P-SiCW. The pretreatment process of excrementum bombycis biowaste was similar to that of silicon cutting waste. The pretreated excrementum bombycis (PEB) is conducive to the interfacial stabilization of the hydrothermal system and serves as the carbon source [18]. During the hydrothermal process, P-SiCW was encapsulated by PEB by virtue of the oxygen-containing groups [29]. Then, the mixture was carbonized under a nitrogen atmosphere at 700 °C for 1 h, and the flake-like porous Si@C composite (FP-Si@C-2) was obtained.

**Figure 1:**
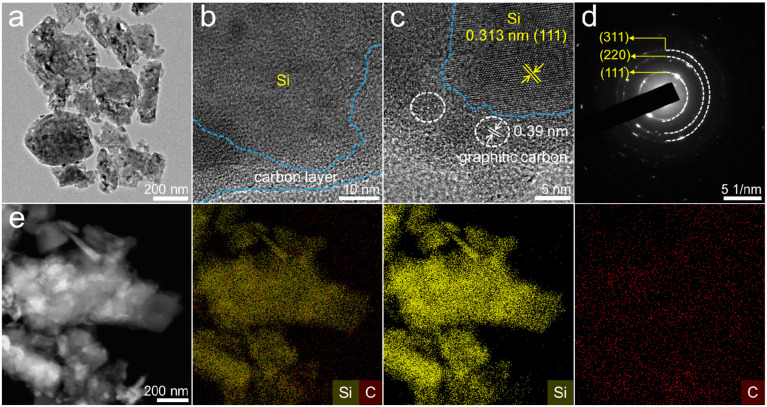
(**a**) TEM image, (**b**,**c**) HRTEM images, (**d**) SAED pattern, and (**e**) EDX elemental mapping of FP-Si@C-2.

The morphological characteristics of the FP-Si@C-2 composite were investigated by scanning electron microscopy (SEM) and transmission electron microscopy (TEM). As shown in Figure 1a and Figure S3b, the FP-Si@C-2 composite is composed of nanoflakes, retaining the morphology of P-SiCW (Figure S2b), but forming abundant pores caused by the pyrolysis of excrementum bombycis. These pores can not only provide buffer spaces to effectively accommodate the volume changes of Si during the lithiation–delithiation process, leading to high structural integrity and an enhanced cycling life, but also facilitate the penetration of electrolyte, contributing to the Li^+^ transfer kinetics and the rate performance [30,31]. To obtain a better understanding of the structure of the FP-Si@C-2 composite, high-resolution TEM (HRTEM) with selected area electron diffraction (SAED) and energy-dispersive X-ray spectroscopy (EDX) were performed. As shown in Figure 1b, Si nanoflakes are wrapped by the graphitic carbon layers derived from the carbonized excrementum bombycis, which would guarantee superior electronic conductivity as well as facile strain relaxation to promote the electron transfer kinetics and provide a more stable solid electrolyte interphase (SEI) layer [32]. More clearly, HRTEM in Figure 1c shows that the lattice spacing of 0.313 nm is consistent with the crystalline Si (111) plane. The crystallinity of Si can be further confirmed by the diffraction spots and rings indexed to the (111), (220), and (311) planes of crystalline Si in the SAED pattern (Figure 1d). In addition, the homogeneous distribution of Si and C as shown in EDX elemental mapping (Figure 1e) is in agreement with the uniform carbon layers coated onto Si nanoflake surfaces, and the weight ratio of Si to C is 83.93:16.07 (Figure S4). The carbon content in FP-Si@C-2 composite further revealed by thermogravimetric analysis (TGA, Figure S5) was calculated as 11.3 wt.%, which is almost consistent with the result of the EDS spectrum in Figure S4. In order to optimize the carbon content, the FP-Si@C-1 and FP-Si@C-3 composites were prepared. Compared with the FP-Si@C-2 composite, the FP-Si@C-1 and FP-Si@C-3 composites display the same morphology (Figure S3a,c) but possess different weight ratios of carbon (8.2 wt.% for FP-Si@C-1 and 24.3 wt.% for FP-Si@C-3, Figure S5). To further study the surface composition of FP-Si@C-2, X-ray photoelectron spectroscopy (XPS) was carried out. The XPS full survey scan in Figure S6a displays Si 2s, Si 2p, and C 1s peaks. The XPS Si 2p spectrum includes three peaks corresponding to Si–Si (99.8 eV), Si–C (100.8 eV), and Si–O (104.6 eV), respectively, showing the presence of pure Si and SiO_2_ (Figure S6b). However, based on the analysis results of the EDS spectrum (Figure S4), the content of SiO_2_ is low. Therefore, the existence of SiO_2_ may be caused by surface oxidation. In addition, the XPS C 1s spectrum is assigned to three peaks at 284.7, 286.2, and 289.0 eV, which are consistent with C–C, C–O, and C=O bonds, respectively (Figure S6c).

**Figure 2:**
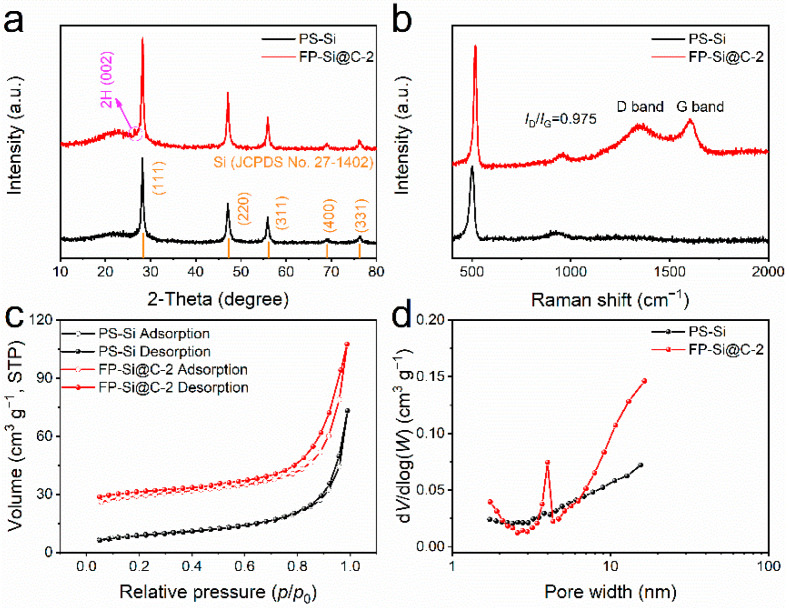
(**a**) XRD patterns, (**b**) Raman spectra, (**c**) N2 adsorption–desorption isotherms, and (**d**) pore size distribution of P-SiCW and FP-Si@C-2.

The phase structures of P-SiCW, FP-Si@C-1, FP-Si@C-2, and FP-Si@C-3 were studied by X-ray diffraction (XRD) analysis, as shown in Figure 2a and Figure S7. Obviously, five distinct diffraction peaks at 28.3°, 47.1°, 56.0°, 69.0°, and 76.0° can be found in all the samples, which are assigned to the (111), (220), (311), (400), and (331) plans of crystalline Si (JCPDS No. 27-1402), respectively. No other peaks are observed in P-SiCW, indicating that the impurities such as residual organics have been effectively treated, which is consistent with the XRF results (Table S1). A broad peak around 22.5° and a feature diffraction peak at 26.4° in the FP-Si@C-1, FP-Si@C-2, and FP-Si@C-3 composites matched with the amorphous carbon and the 2H(002) plane of graphite, respectively, indicating their disordered structures with a low graphitization degree [33]. To gain better insight into the structures of carbon in the FP-Si@C composites, Raman spectroscopy was carried out (Figure 2b and Figure S8). Besides a sharp crystalline Si peak around 518 cm^−1^, there are two characteristic peaks of carbon at 1350 cm^−1^ and 1590 cm^−1^, corresponding to the D band assigned to the disordered/defective carbon with graphitic sp^3^-hybridization and the G band associated with the graphitic carbon derived from the E_2g_ vibratory mode of sp^2^ bond, respectively [34]. Moreover, the intensity ratio of the D and G peak (*I*_D_/*I*_G_) can be applied to evaluate the graphitization degree of carbonaceous materials [35]. The *I*_D_/*I*_G_ values for FP-Si@C-1 FP-Si@C-2, and FP-Si@C-3 were calculated as 0.947, 0.975, and 0.959, respectively, where the decreased ratio suggests an enhanced graphitization degree, thereby resulting in strengthened electrical conductivity to boost the Li^+^ transport.

Due to the addition of slightly graphitic carbon, the type of N_2_ adsorption–desorption curves for the FP-Si@C-2 composite is type IV (Figure 2c), suggesting the existence of meso-macropores on the FP-Si@C-2 surface (Figure 2d). The meso-macroporous structure could alleviate the morphology changes derived from the large volume changes of Si by ensuring the structural integrity and strain relaxation during the discharging/charging process, thus enhancing the cycling stability [30,31,36]. Additionally, the Brunauer–Emmett–Teller (BET) specific surface area (SSA) of the FP-Si@C-2 composite is decreased from 113.50 m^2^ g^−1^ of the P-SiCW to 61.76 m^2^ g^−1^, and the pore volume of the P-SiCW and FP-Si@C-2 is 0.24 and 0.14 cm^3^ g^−1^, respectively (Table S2), which is conducive to reducing the side reactions, consequently resulting in a promoted Coulombic efficiency (CE) [14].

**Figure 3:**
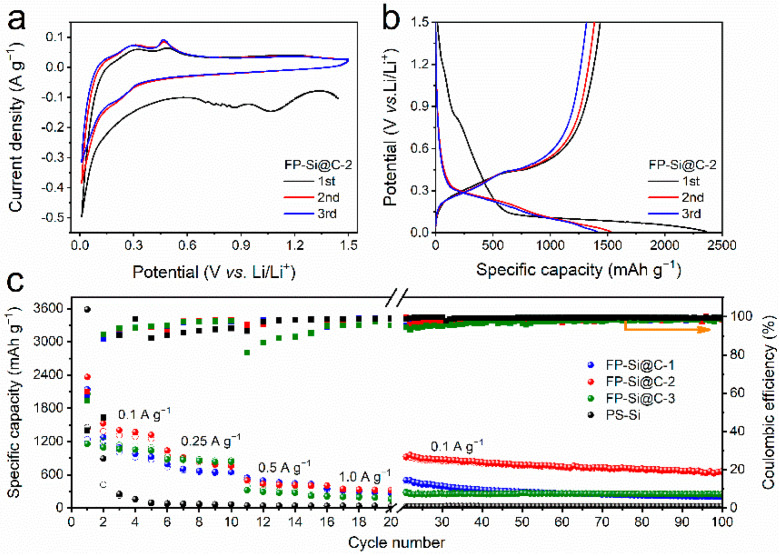
Electrochemical Li+ storage performance of Si-based anodes in half-cells. (**a**) The initial three CV curves of FP-Si@C-2 at 0.1 mV s^−1^; (**b**) initial three GCD curves of FP-Si@C-2 at 0.1 A g^−1^; (**c**) rate capability and long-term cycling stability of P-SiCW, FP-Si@C-1, FP-Si@C-2, and FP-Si@C-3.

To investigate the electrochemical Li^+^ storage performance of FP-Si@C composite in detail, half-cells were assembled employing 1.0 M LiPF_6_ in ethylene carbonate (EC)/dimethyl carbonate (DMC) (1:1 in volume ratio) as the electrolyte. Figure 3a shows the first three cyclic voltammetry (CV) curves of the FP-Si@C-2 composite tested in a potential range of 0.01–1.50 V (vs. Li/Li^+^) at a scan rate of 0.1 mV s^−1^. In the first cathodic scan, two broad reduction bumps at 0.86 and 1.07 V correspond to the irreversible reactions between Li^+^ and surface functional groups of Si, and the SEI formation, respectively, and then vanish in the subsequent cycles [37]. Another reduction peak observed below 0.10 V is attributed to the Li_x_Si formation, while two oxidation peaks around 0.31 and 0.49 V are assigned to the dealloying stage of Li_x_Si [37]. In the latter cycles, obviously increased intensities of the two oxidation peaks are found due to the activation process of FP-Si@C-2. Moreover, a new reduction peak at 0.21 V is detected, which can be ascribed to the alloying process of Si [38]. Importantly, the CV curves almost overlap after the second cycle, indicating the high electrochemical reversibility of FP-Si@C-2. The CV profiles of all the FP-Si@C composites are similar to that of P-SiCW, illustrating the electrochemical characteristic of Si-based materials (Figure 3a and Figure S9a–c). Figure 3b and Figure S9d–f exhibit the galvanostatic charge–discharge (GCD) profiles of all the FP-Si@C composites and P-SiCW for the initial three cycles measured at a current density of 0.1 A g^−1^ between 0.01 and 1.50 V (vs. Li/Li^+^). The first discharge/charge specific capacity of P-SiCW, FP-Si@C-1, FP-Si@C-2, and FP-Si@C-3 is 3581/1451, 2139/1238, 2364/1436, and 2059/1158 mAh g^−1^, with an initial Coulombic efficiency (ICE) of 40.5%, 57.9%, 60.7%, and 56.2%, respectively, suggesting that the addition of a moderate amount of slightly graphitic carbon could decrease the amount of “dead lithium” by increasing the electronic conductivity and suppressing the fracture of Si; hence, the FP-Si@C-2 composite delivers a higher ICE [14]. Meanwhile, the CE of FP-Si@C-2 composite can reach 93.7% at the third cycle, indicating high Li^+^ storage reversibility. Therefore, as depicted in Figure 3c, the cycling stability and CE of all the FP-Si@C composites and P-SiCW were further compared and investigated at a current density of 0.1 A g^−1^. Although no discharge/charge-specific capacity can be found for the P-SiCW with low electrical conductivity, the addition of slightly graphitic carbon noticeably enhances the Li^+^ storage capability of the pure Si. Meanwhile, it is worth noting that the corresponding capacity retention is obviously promoted by increasing the amount of slightly graphitic carbon. The FP-Si@C-2 composite retains a high specific capacity of 654 mAh g^−1^ with nearly 99.0% CE after 100 cycles, which is due to the fact that the meso-macroporous structure and the protective carbon layer can accommodate the volume changes of Si during the alloying–dealloying process [30,31,32,36]. The rate performance also has an important impact on the practical applications of anode materials for Li^+^ batteries. As shown in Figure 3c, the FP-Si@C-2 composite exhibits a high reversible capacity of 406 mAh g^−1^ at 1.0 A g^−1^, where 1322 mAh g^−1^ of the specific capacity at 0.1 A g^−1^ is maintained. Note that when the current density is switched back to 0.1 A g^−1^, the specific capacity raises to 924 mAh g^−1^. In sharp comparison, the discharge capacity of FP-Si@C-2 composite is higher than that of P-SiCW at each current density.

**Figure 4:**
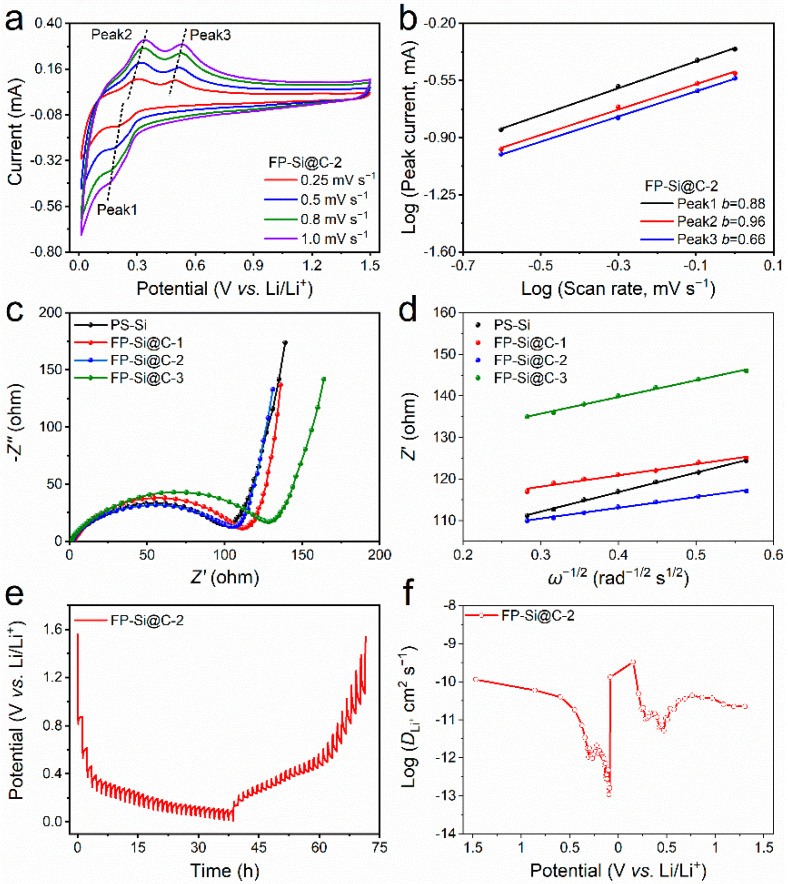
Kinetic analysis of Si-based anodes. (**a**) The CV curves of FP-Si@C-2 at different scan rates; (**b**) The log(*i*)–log(*v*) plots of FP-Si@C-2; (**c**) electrochemical impedance spectra of P-SiCW, FP-Si@C-1, FP-Si@C-2, and FP-Si@C-3; (**d**) fitting *Z′* and *ω*^−1/2^ of P-SiCW, FP-Si@C-1, FP-Si@C-2, and FP-Si@C-3; (**e**) GITT profiles of FP-Si@C-2; (**f**) Li+ diffusion coefficients of FP-Si@C-2.

To investigate the Li^+^ storage kinetics in-depth, the CV curves of all the FP-Si@C composites and P-SiCW at different scan rates from 0.25 to 1.0 mV s^−1^ were recorded (Figure 4a and Figure S10a–c). Obviously, the CV shapes of all the samples are similar even at a high sweep speed, including one cathodic peak and two anodic peaks. In addition, the relationship between the peak currents (*i*) and the sweep rates (*v*) obeys the following equation [39]:

*i* = *av^b^* (1)

log(*i*) = *b*log(*v*) + log(*a*) (2)

where *a* and *b* are the adjustable constants, while the b value can be calculated based on the slope of log(*i*)–log(*v*). The *b* value of 0.5 or 1.0 indicates the diffusion-controlled process or the capacitance-limited mechanism for Li^+^ storage behaviors, respectively. As shown in Figure 4b, the *b* values of peak 1, peak 2, and peak 3 for FP-Si@C-2 composite are 0.82, 0.78, and 0.77, respectively, implying the Li^+^ storage dynamics are dominated by the diffusion control and the surface capacitive behavior simultaneously. In comparison, the *b* values of P-SiCW, FP-Si@C-1, and FP-Si@C-3 are displayed in Figure S10d–f, respectively, which demonstrates that the FP-Si@C-2 composite exhibits improved kinetics. To get an insight into the Li^+^ diffusion kinetics and electrical resistance of the FP-Si@C-2 composite, electrochemical impedance spectroscopy (EIS) measurements were carried out from 100 kHz to 10 MHz As represented in Figure 4c, the Nyquist plots of all the FP-Si@C composites and P-SiCW were fitted utilizing the equivalent circuit (Figure S11), including an intercept with the *Z′*-axis at the high-frequency range related to the solution resistance (*R*_s_) between the electrolyte and separator, a midfrequency semicircle associated with the charge transfer resistance (*R*_ct_) between the electrolyte and electrode materials, and a straight line at low frequency involved in the Li^+^ diffusion impedance into the active layer, named as the Warburg impedance (*W*) [40]. The *R*_ct_ and Warburg values of all the samples are summarized in Table S3. Compared with the *R*_ct_ value of P-SiCW (97.8 ohm), FP-Si@C-1 (102.8 ohm), and FP-Si@C-3 (116.5 ohm), the FP-Si@C-2 composite delivers the lowest *R*_ct_ value (91.5 ohm), clarifying that the addition of an appropriate amount of slightly graphitic carbon facilitates the interfacial charge transfer. Additionally, the Li^+^ diffusion coefficient (*D*_Li_^+^) can be obtained by fitting *Z′* and *ω*^−1/2^ based on the following equations [39]:

*Z′* = *R_e_* + *R_ct_* + *σω*^−1/2^ (3)

(4) $${D_{\mathrm{Li}}}^{+}=\frac{R^{2}T^{2}}{2A^{2}n^{4}F^{4}C^{2}\sigma^{2}}$$

where *σ*, *ω*, *R*, *T*, *A*, *n*, *F*, and *C* stand for the Warburg factor, angular frequency, gas constant, absolute temperature, electrode surface area, transfer electron number per molecule, Faraday constant, and Li^+^ molar concentration, respectively. As illustrated in Figure 4d, the *σ* value of the P-SiCW, FP-Si@C-1, FP-Si@C-2, and FP-Si@C-3 is 46.9, 27.2, 25.9, and 40.2, respectively. The *D*_Li_^+^ of FP-Si@C-2 is 3.28 times higher than that of P-SiCW, indicating that FP-Si@C-2 composite possesses much stronger interface kinetics. The *D*_Li_^+^ value can be further evaluated by the galvanostatic intermittent titration technique (GITT) with a pulse current of 0.1 A g^−1^ for a pulse time of 10 min between 1 h rest intervals (Figure 4e) and quantitatively determined based on Fick’s second law [39]:

(5) $$D_{{\mathrm{Li}}^{+}}=\frac{4}{\pi \tau }{\left(\frac{m_{B}V_{M}}{M_{B}S}\right)}^{2}{\left(\frac{\Delta E_{S}}{\Delta E_{\tau }}\right)}^{2}$$

where *τ*, *m*_B_, *M*_B_, *V*_M_, and *S* represent the constant current time, active mass, molar mass, molar volume, and active surface area, respectively. ∆*E*_S_ and ∆*E*_τ_ mean the voltage change between steps and the voltage varying between pulse time, respectively (Figure S12). As shown in Figure 4f, the *D*_Li_^+^ change of FP-Si@C-2 composite during the alloying and dealloying process displays a “W”-type profile with two minimum ranges associated with the strong attractions between the Li^+^ and active materials, or some ordered–disordered transitions [41,42]. Notably, the *D*_Li_^+^ value is comparatively high at the start of lithiation, which can be ascribed to the coating of slightly graphitic carbon on the surface of Si facilitating the Li^+^ into the host matrix [18]. As a result, the *D*_Li_^+^ value of FP-Si@C-2 composite (10^−9.5^–10^−13^ cm^2^ s^−1^) is 100 times higher than that of P-SiCW [41,42], implying faster Li^+^ diffusion kinetics in the FP-Si@C-2 composite. By virtue of the above discussion, the better cycling and rate performance of FP-Si@C-2 composite are attributable to its unique structural features: (i) the meso-macroporous structure and the protective carbon layer can accommodate the volume changes of Si during the alloying–dealloying process; (ii) the meso-macroporous structure also can facilitate the electrolyte penetration and the ion transport, leading to fast Li^+^ transfer kinetics during the discharge–charge process; (iii) the addition of slightly graphitic carbon with high electrical conductivity is conducive to rapid Li^+^ diffusion in/out of the active materials. Therefore, the introduction of slightly graphitic carbon is conducive to enhancing the electrochemical performance of pure Si, which is also an effective means of endowing other negative electrodes with overall structural integrity and fast electrochemical kinetics for superb Li^+^ storage.

## 4. Conclusions

To summarize, this work develops a hydrothermal approach accompanied by heat treatment for the synthesis of flake-like porous Si@C (FP-Si@C) composite anode materials for LIBs using photovoltaic Si cutting waste and biomass excrementum bombycis. The abundant pores in FP-Si@C composite can not only provide efficient buffers for accommodating the large volume changes of Si during the lithiation/delithiation process but also facilitate the electrolyte penetration. At the same time, Si nanoflakes are wrapped by the slightly graphitic carbon layer with high mechanical strength and electrical conductivity derived from the carbonized excrementum bombycis, which is beneficial to maintaining the electrode integrity and facilitating the electron/ion transfer. By integrating these superiorities, the FP-Si@C composite exhibits excellent lithium storage performance, including a high discharge capacity of 1322 mAh g^−1^ at 0.1 A g^−1^, remarkable cycle stability with capacity retention of 70.8% after 100 cycles, and a superior rate capability of 406 mAh g^−1^ at 1.0 A g^−1^. Hence, this work not only marks a significant step over the high-value application of photovoltaic Si cutting waste but also exploits a novel route to the design of alloying-type anodes for energy storage.

## Data Availability

Not applicable.

## Supplementary Materials

The following supporting information can be downloaded at: https://www.mdpi.com/article/10.3390/nano12162875/s1, Figure S1: Schematic illustration of the synthesis process for FP-Si@C composites; Figure S2: SEM images of (a) silicon cutting waste scragglomerations and (b) P-SiCW; Figure S3: SEM images of (a) FP-Si@C-1, (b) FP-Si@C-2, and (c) FP-Si@C-3; Figure S4: EDS spectrum of FP-Si@C-2 (Inset is the weight and atomic content of C and Si); Figure S5: TGA curves of P-SiCW, PEB, FP-Si@C-1, FP-Si@C-2, and FP-Si@C-3; Figure S6: (a) XPS full spectrum, (b) XPS Si 2p spectrum, and (c) XPS C 1s spectrum of FP-Si@C-2; Figure S7: XRD patterns of FP-Si@C-1 and FP-Si@C-3; Figure S8: Raman spectra of FP-Si@C-1 and FP-Si@C-3; Figure S9: (a–c) The initial three CV curves of P-SiCW, FP-Si@C-1, and FP-Si@C-3 at 0.1 mV s^−1^. (d–f) The initial three GCD curves of P-SiCW, FP-Si@C-1, and FP-Si@C-3 at 0.1 A g^−1^; Figure S10: (a–c) The CV curves of P-SiCW, FP-Si@C-1, and FP-Si@C-3 at different scan rates. (d–f) The log(*i*)-log(*v*) plots of P-SiCW, FP-Si@C-1, and FP-Si@C-3; Figure S11: Equivalent circuit model linked with EIS curves; Figure S12: Partial enlarged view about GITT results and marked ∆*E*_S_ and ∆*E*_τ_ of FP-Si@C-2; Table S1: XRF results of silicon cutting waste and P-SiCW; Table S2: BET specific surface area, pore volume, and BJH pore size of P-SiCW and FP-Si@C-2; Table S3: The *R*_ct_ and Warburg values of P-SiCW, FP-Si@C-1, FP-Si@C-2 and FP-Si@C-3.
