# Supplementary material for: High-Value Utilization of Silicon Cutting Waste and Excrementum Bombycis to Synthesize Silicon–Carbon Composites as Anode Materials for Li-Ion Batteries
Source: Nanomaterials (Basel). 2022 Aug 21;12(16):2875. doi: 10.3390/nano12162875 (PMC9415209; doi:10.3390/nano12162875)
Supplement: Supplementary file 1 [file nanomaterials-12-02875-s001.zip › nanomaterials-1844653-supplementary.pdf]

Supplementary Materials

For

High-Value Utilization of Silicon Cutting Waste and Excrementum Bombycis to Synthesize Silicon–Carbon Composites as Anode Materials for Li-Ion Batteries

Hengsong Ji [1,†], Jun Li [1,†], Sheng Li [1], Yingxue Cui [1,*], Zhijin Liu [1], Minggang Huang [2], Chun Xu [2], Guochun Li [1], Yan Zhao [3] and Huaming Li [1]

[1] Institute for Energy Research, Jiangsu University, Zhenjiang 212013, China;
[2] Key Laboratory of Fine Chemical Application Technology of Luzhou, Luzhou 646099, China
[3] College of Chemistry and Chemical Engineering, Inner Mongolia University, Hohhot 010021, China
* Correspondence: yxcui@ujs.edu.cn (Y.C.)
† These authors contributed equally to this work.

Supplementary Figures:

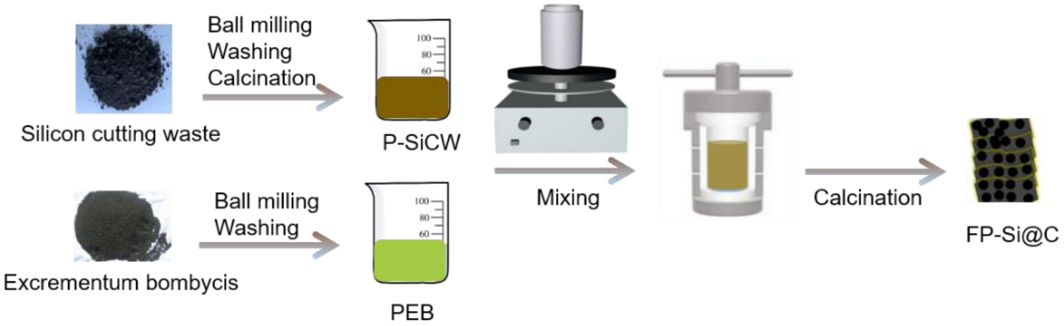

Figure S1. Schematic illustration of the synthesis process for FP-Si@C composites.

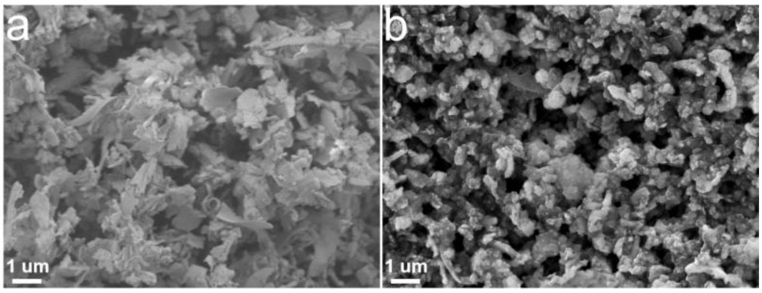

Figure S2. SEM images of (**a**) silicon cutting waste scragglomerations and (**b**) P-SiCW.

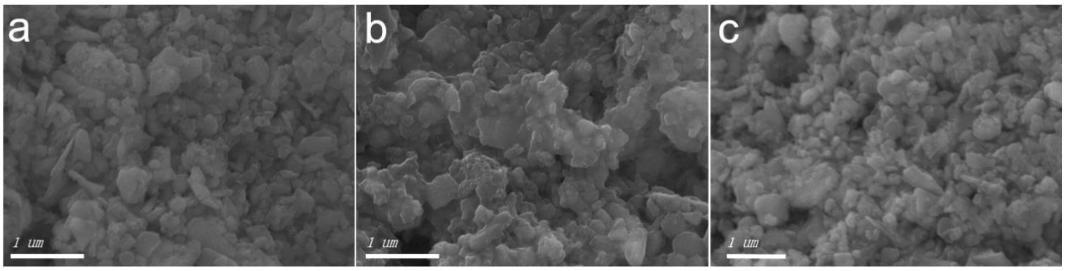

Figure S3. SEM images of (**a**) FP-Si@C-1, (**b**) FP-Si@C-2, and (**c**) FP-Si@C-3.

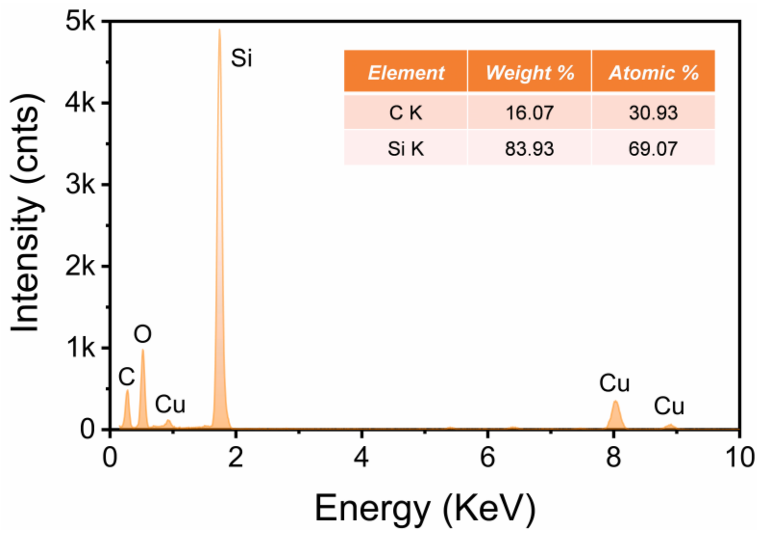

Figure S4. EDS spectrum of FP-Si@C-2 (Inset is the weight and atomic content of C and Si).

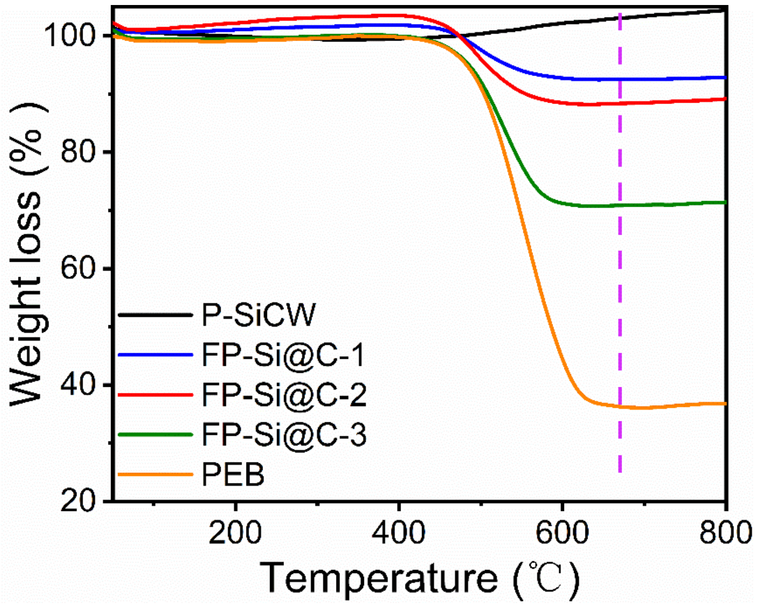

Figure S5. TGA curves of P-SiCW, PEB, FP-Si@C-1, FP-Si@C-2, and FP-Si@C-3.

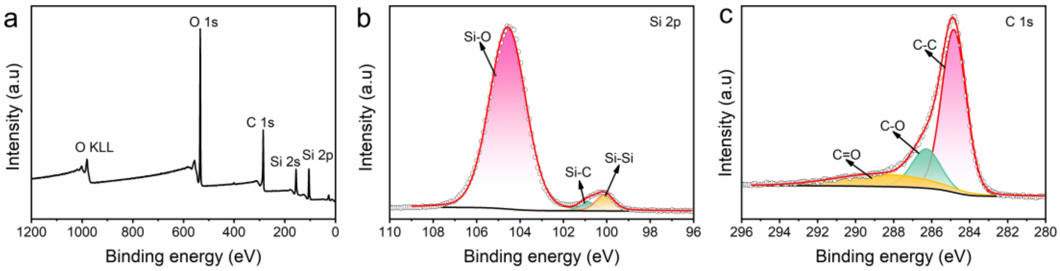

Figure S6. (**a**) XPS full spectrum, (**b**) XPS Si 2p spectrum, and (**c**) XPS C 1s spectrum of FP-Si@C-2.

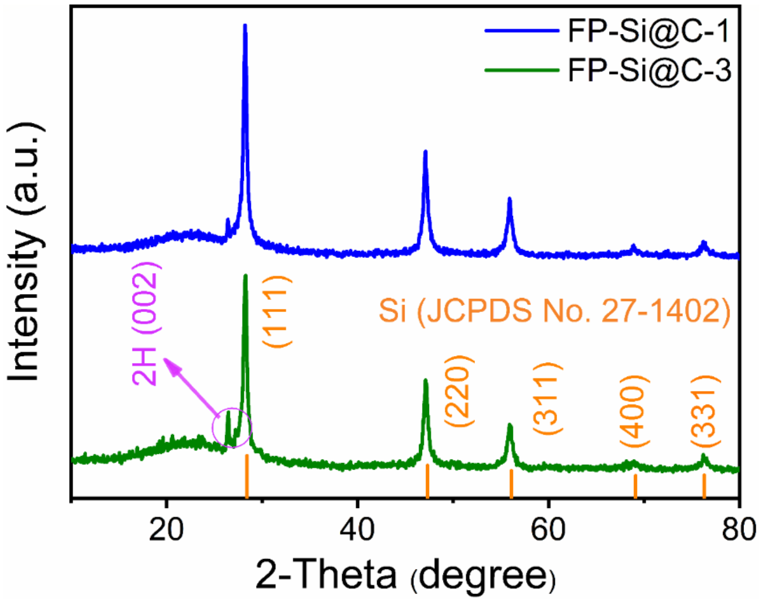

Figure S7. XRD patterns of FP-Si@C-1 and FP-Si@C-3.

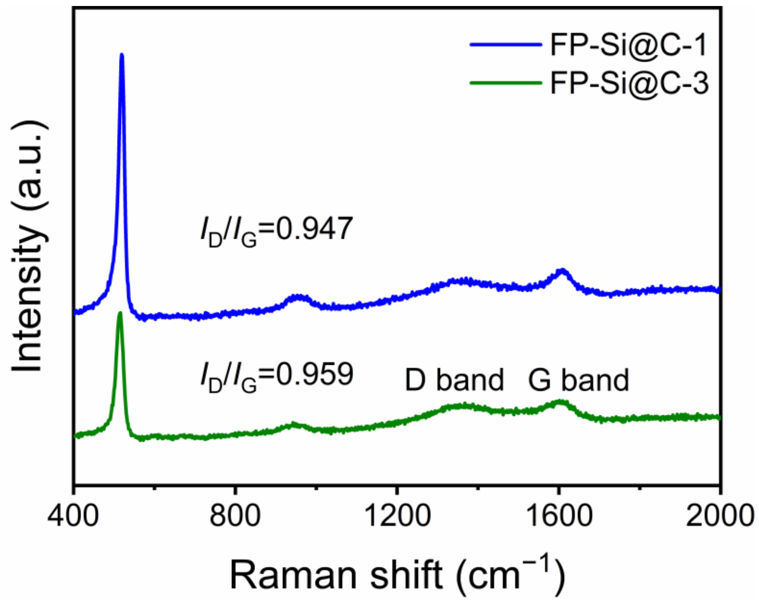

Figure S8. Raman spectra of FP-Si@C-1 and FP-Si@C-3.

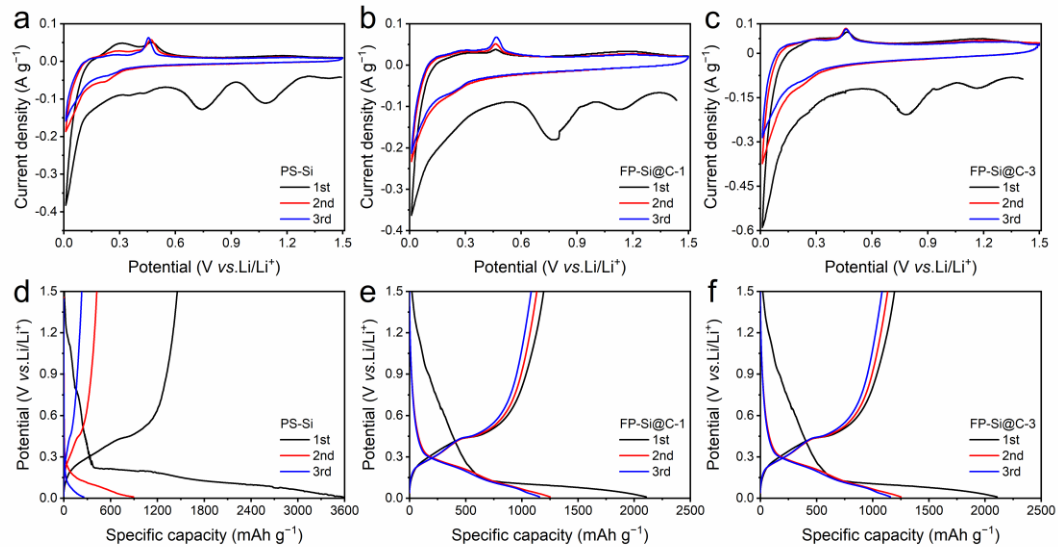

Figure S9. (**a–c**) The initial three CV curves of P-SiCW, FP-Si@C-1, and FP-Si@C-3 at 0.1 mV s^{-1}. (**d–f**) The initial three GCD curves of P-SiCW, FP-Si@C-1, and FP-Si@C-3 at 0.1 A g^{-1}.

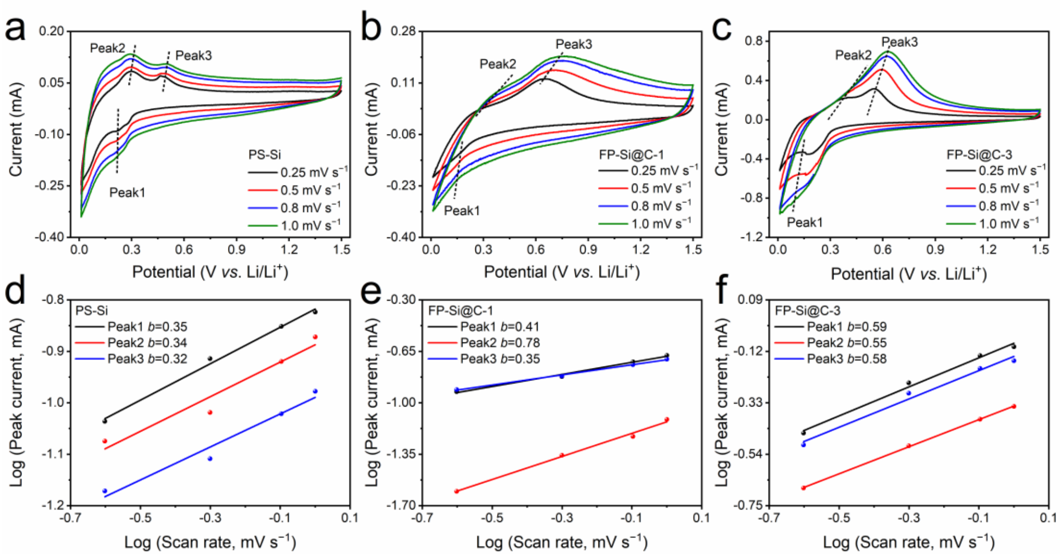

Figure S10. (**a–c**) The CV curves of P-SiCW, FP-Si@C-1, and FP-Si@C-3 at different scan rates. (**d–f**) The log(*i*)-log(*v*) plots of P-SiCW, FP-Si@C-1, and FP-Si@C-3.

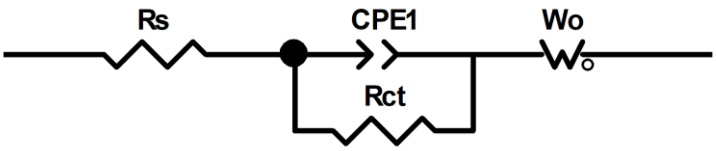

Figure S11. Equivalent circuit model linked with EIS curves.

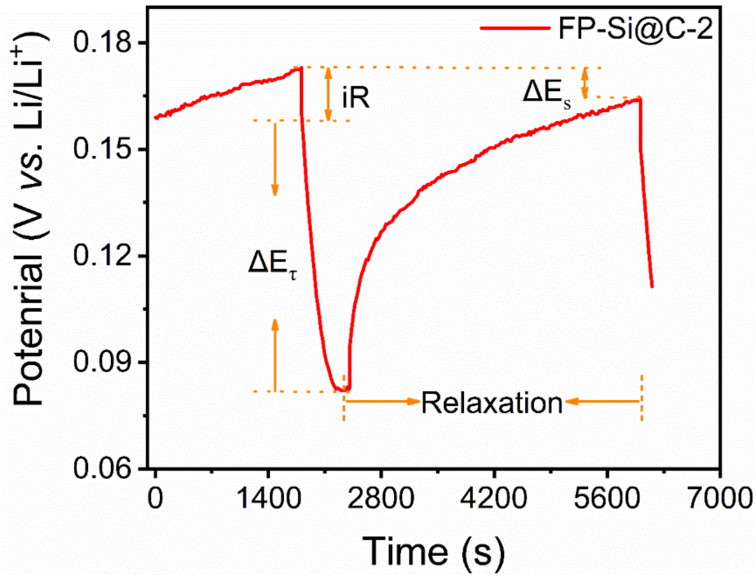

Figure S12. Partial enlarged view about GITT results and marked ΔE_S and ΔE_τ of FP-Si@C-2.

Supplementary Tables:

Table S1. XRF results of silicon cutting waste and P-SiCW.

Sample EA		Si	Fe	Cr	Ni	Cu	Cl	Mg	Mo
**Weight** （%）	Silicon cutting waste	83.84	10.34	3.44	1.8	0.134	0.0637	0.0382	0.037
	P-SiCW	99.83	0.0067	0.0007	0.0256	0.0005	0.0027	0.0336	0.0043

Table S2. BET specific surface area, pore volume, and BJH pore size of P-SiCW and FP-Si@C-2.

Sample	S_{BET} (m^2 g^{-1})	V_{total} (cm^3 g^{-1})	Average pore size (BJH) (nm)
P-SiCW	113.50	0.24	8.31
FP-Si@C-2	61.76	0.14	9.32

Table S3. The R_{ct} and Warburg values of P-SiCW, FP-Si@C-1, FP-Si@C-2 and FP-Si@C-3.

Sample	R_{ct} (ohm)	σ (Ω $S^{-1/2}$)
P-SiCW	97.8	46.9
FP-Si@C-1	102.8	27.2
FP-Si@C-2	91.5	25.9
FP-Si@C-3	116.5	40.2
